# Supplementary figures and images for: The seasonality of infections in tropical Far North Queensland, Australia: A 21-year retrospective evaluation of the seasonal patterns of six endemic pathogens
Source: PLOS Glob Public Health. 2022 May 25;2(5):e0000506. doi: 10.1371/journal.pgph.0000506 (PMC10021965; doi:10.1371/journal.pgph.0000506)

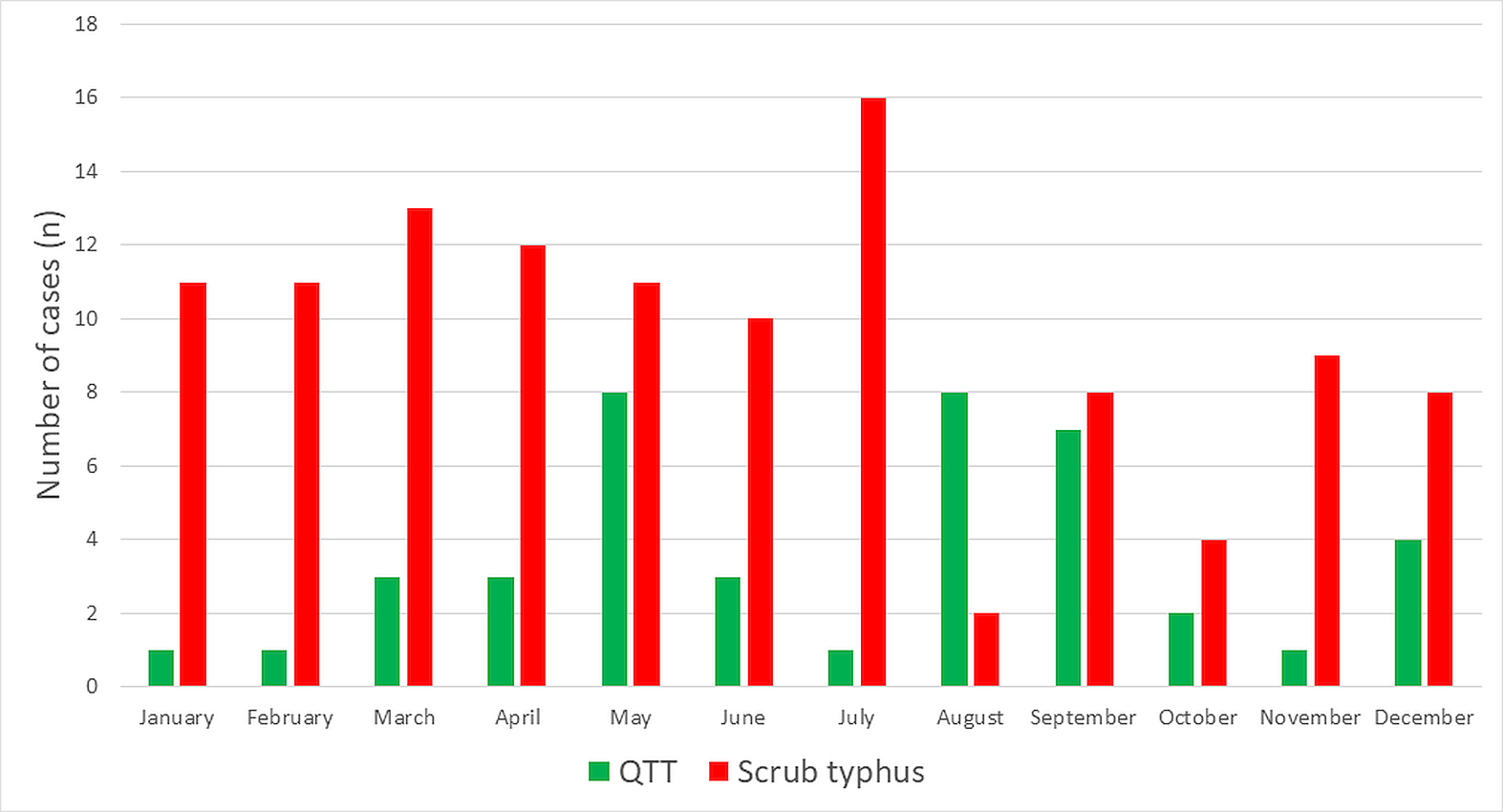

Supplement: S1 Fig — (TIF) [file pgph.0000506.s001.tif]

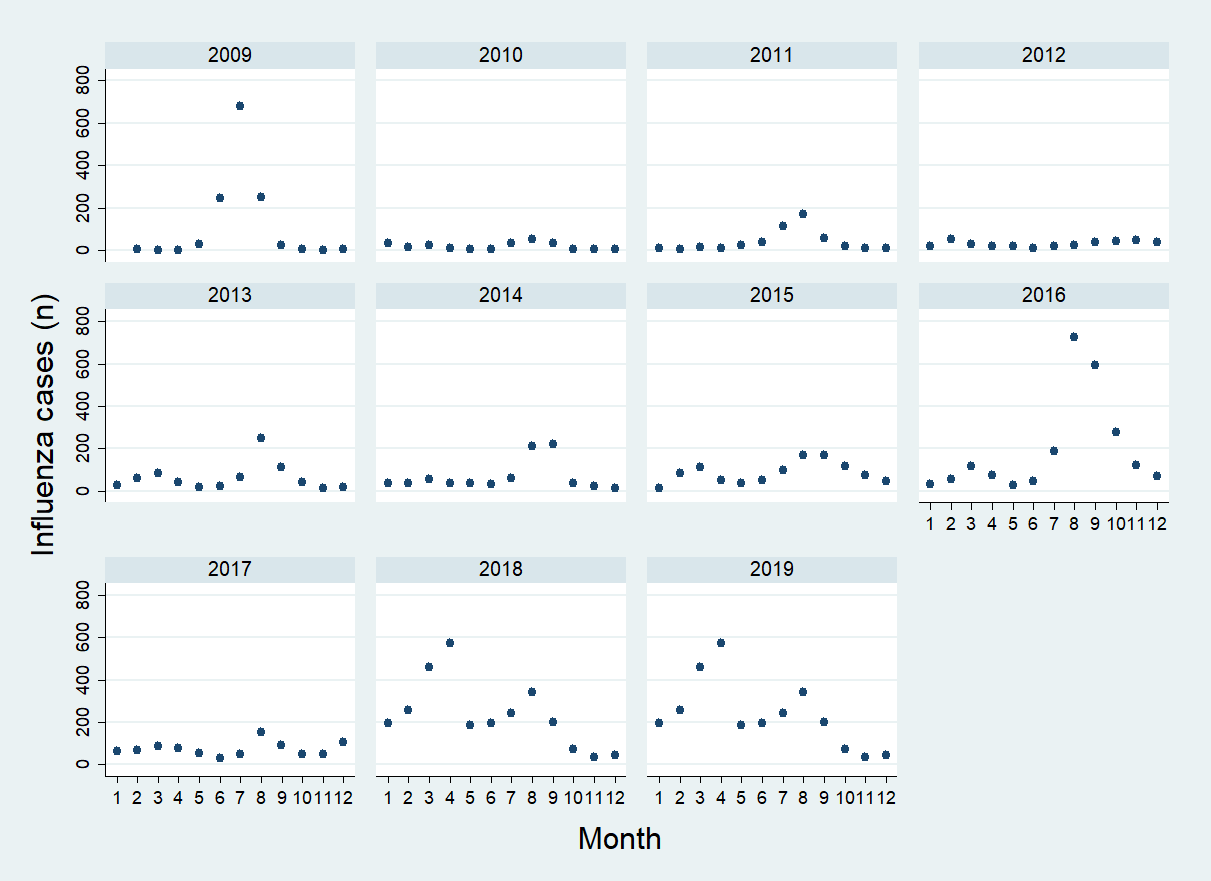

Supplement: S2 Fig — Only data after 2008 are presented as data collection prior to 2009 were incomplete. (TIF) [file pgph.0000506.s002.tif]
