# Supplementary material for: The seasonality of infections in tropical Far North Queensland, Australia: A 21-year retrospective evaluation of the seasonal patterns of six endemic pathogens
Source: PLOS Glob Public Health. 2022 May 25;2(5):e0000506. doi: 10.1371/journal.pgph.0000506 (PMC10021965; doi:10.1371/journal.pgph.0000506)
Supplement: S1 Table — (DOCX) [file pgph.0000506.s003.docx]

**S1 Table. Correlation between individual climatic variables, Cairns, Far North Queensland, 2001-2019.**

|  | Temperature | | rainfall | | Clouds | | Humidity | | Dew point | |
| --- | --- | --- | --- | --- | --- | --- | --- | --- | --- | --- |
|  | r_s_ | p | r_s_ | p | r_s_ | p | r_s_ | p | r_s_ | p |
| Temperature | - | - | <0.0001 | <0.0001 | 0.15 | 0.01 | -0.05 | 0.42 | 0.70 | <0.0001 |
| Rainfall | 0.49 | <0.0001 | - | - | 0.26 | <0.0001 | 0.44 | <0.0001 | 0.77 | <0.0001 |
| Clouds | 0.15 | 0.01 | 0.26 | <0.0001 | - | - | 0.19 | 0.02 | 0.25 | <0.0001 |
| Humidity | -0.05 | 0.42 | 0.44 | <0.0001 | 0.19 | 0.02 | - | - | 0.58 | <0.0001 |
| Dew point | 0.70 | <0.0001 | 0.77 | <0.0001 | 0.25 | <0.0001 | 0.58 | <0.0001 | - | - |

Analysed using Spearman’s rho (r_s_)
