# Supplementary material for: The seasonality of infections in tropical Far North Queensland, Australia: A 21-year retrospective evaluation of the seasonal patterns of six endemic pathogens
Source: PLOS Glob Public Health. 2022 May 25;2(5):e0000506. doi: 10.1371/journal.pgph.0000506 (PMC10021965; doi:10.1371/journal.pgph.0000506)
Supplement: Attachment — Submitted filename: PGPH-D-22-00139 R1 Response to reviewers.docx [file pgph.0000506.s005.docx]

**Response to reviewers**

We thank the Editorial staff and the reviewers for the time that they have taken to review our manuscript and the very helpful comments that they have made to improve the work. Please find below our point-by-point responses to their comments. For clarity, the reviewers’ comments are presented in blue normal text, while our responses are in black normal text.

Reviewers' comments:

Reviewer's Responses to Questions

**Comments to the Author**

1. Does this manuscript meet PLOS Global Public Health’s [publication criteria](https://journals.plos.org/globalpublichealth/s/criteria-for-publication)? Is the manuscript technically sound, and do the data support the conclusions? The manuscript must describe methodologically and ethically rigorous research with conclusions that are appropriately drawn based on the data presented.

Reviewer #1: Yes

Reviewer #2: Yes

**Response:** We thank the reviewers for the time that they have taken to review the manuscript and are pleased that they feel that the manuscript meets PLOS Global Public Health’s [publication criteria](https://journals.plos.org/globalpublichealth/s/criteria-for-publication).

2. Has the statistical analysis been performed appropriately and rigorously?

Reviewer #1: Yes

Reviewer #2: I don't know

**Response:** We thank the reviewers for the time that they have taken to review the manuscript and are pleased that Reviewer 1 is satisfied with our statistical analysis. One of our co-authors, Professor Matthew Law, a biostatistician and Head of the Biostatistics and Databases Program at the Kirby Institute, UNSW Sydney, oversaw the statistical analysis in the paper.

3. Have the authors made all data underlying the findings in their manuscript fully available (please refer to the Data Availability Statement at the start of the manuscript PDF file)?

The [PLOS Data policy](https://journals.plos.org/globalpublichealth/s/data-availability) requires authors to make all data underlying the findings described in their manuscript fully available without restriction, with rare exception. The data should be provided as part of the manuscript or its supporting information, or deposited to a public repository. For example, in addition to summary statistics, the data points behind means, medians and variance measures should be available. If there are restrictions on publicly sharing data—e.g. participant privacy or use of data from a third party—those must be specified.

Reviewer #1: Yes

Reviewer #2: Yes

**Response:** We thank the reviewers for the time that they have taken to review the manuscript and are pleased that they are happy with the data availability.

4. Is the manuscript presented in an intelligible fashion and written in standard English?

PLOS Global Public Health does not copyedit accepted manuscripts, so the language in submitted articles must be clear, correct, and unambiguous. Any typographical or grammatical errors should be corrected at revision, so please note any specific errors here.

Reviewer #1: Yes

Reviewer #2: Yes

**Response:** We thank the reviewers for the time that they have taken to review the manuscript and are pleased that they are happy that the paper is written intelligibly.

5. Review Comments to the Author

Please use the space provided to explain your answers to the questions above. You may also include additional comments for the author, including concerns about dual publication, research ethics, or publication ethics. (Please upload your review as an attachment if it exceeds 20,000 characters)

Reviewer #1: This study examined seasonality with data accumulated over a long period of 21 years. Although it shows very interesting results, the logical development to draw conclusions is insufficient. It is recommended to supplement this point and reflect it in the mabuscript. On the other hand, the manuscript is very well written in an easy to read English style.

**Response:** We thank the reviewers for the time that they have taken to review the manuscript and are happy that they feel it is well written. We have revised the manuscript extensively and hopefully the Reviewer and Editorial staff now feel that it the paper develops our arguments more logically.

The conclusion of the paper is that although anthropogenic climate change poses an existential threat to life on the planet and will, almost certainly impact on the incidence of infectious diseases in the future, in this region of tropical Australia other environmental, behavioural, and societal factors have an important - and presently greater - impact on disease incidence. It is essential to consider these factors when devising local public health strategies to reduce the morbidity and mortality of the highlighted diseases in this tropical region (lines 44-51 and lines 405-412).

Over the course of the study, there was no significant change in key climactic factors or the incidence of severe weather events (table 1), however, we show the incidence of melioidosis and rickettsial diseases have increased markedly in the region over the same period (figure 2).

This increasing incidence does not appear to be explained by improvements in case ascertainment, instead the incidence of both diseases appears more likely to be explained by the significant urban expansion that occurred during the study period. The construction of a large motorway to the south of Cairns increased the local incidence of melioidosis in the urban Cairns region tenfold (lines 277-281). Meanwhile the 10-fold rise in the incidence of rickettsial diseases is likely to be at least partially explained by a >32% rise in population in the region and expansion of the urban-rural fringe and increased interactions with tick and mite habitats (lines 312-314).

Although there was no significant increase in the incidence of the other highlighted diseases, we emphasise the importance of human and societal factors in the incidence of other diseases. Leptospirosis was, unsurprisingly, highly seasonal, however, approximately 85% of cases occurred in individuals with potential occupational exposure (lines 301-308). The burden of *Staphylococcus aureus* blood stream infections was disproportionately borne by the local Indigenous Australian population, many of whom suffer from significant socioeconomic disadvantage (lines 282-290 and lines 347-351). The interseasonality of influenza in the region is influenced by the region’s reputation as an international tourist destination, with influenza likely to be introduced by travellers escaping the Northern Hemisphere’s winter (lines 355-359). The impact of these infections might be mitigated by improving occupational health standards, adjusting the timing of vaccination, and addressing the underlying social inequity that drives the incidence of many infectious diseases and poor health outcomes generally.

Previous studies have highlighted the role that climate factors play in all the diseases highlighted in this paper. We do not dispute those associations. While anthropogenic climate change poses a threat to the world (and to tropical regions in particular) and may influence the burden of these infections in the future, our study highlights the fact that, even for seasonal diseases, other factors presently have a greater effect on disease incidence. Public health strategies must also target these broader drivers of infection if they are to be effective.

Lines 37-39 (Abstract) : Seasonality is most affected by temperature and humidity. And the most important factors that determine climate are temperature and humidity. Therefore, it is necessary to define specifically what environmental, human, and climatic factors mean.

**Response:** We agree that is important to define our terms carefully. However, definitions are best presented in the text of the paper rather than the abstract, given the word count limits. However, we have expanded our discussion on these factors in the introduction (lines 83-90 and lines 131-162)

(Introduction) The meaning of seasonality, the characteristics of the study region, the characteristics of the diseases to be studied, and why this diseases were chosen should be logically developed.

**Response:** This is a very fair comment. We have added a definition of seasonality (lines 69-70).

We provided a relatively detailed description of the study region in the initial submission, but we have expanded this further to discuss other aspects of the region including more information about the geography, the population, the landscape, and the economy. We have discussed particularly, how these factors contribute to the incidence of infectious diseases in the region (lines 91-164). We have also added a figure to highlight the precise geographical location of the region and its proximity to Papua New Guinea for the international readership. We would be happy to add additional information if the Editorial staff felt that this needed more development.

We did, in fact, describe why these diseases were chosen in lines 108-109 of the methods of the previous submission. However, we have now added a sentence about why these diseases were chosen in the introduction as well (lines 139-164). Leptospirosis, melioidosis, cryptococcus and rickettsial diseases are the predominant tropical pathogens encountered in the region. *Staphylococcus aureus* and influenza were included as comparators. Influenza is perhaps the archetypal seasonal disease (although as we discuss in the paper the seasonality is more complex in tropical regions). Meanwhile, *S. aureus* was chosen as a comparator as this might also have been anticipated to show seasonal variation (as has been reported in previous studies - eg Leekha et al Clin Microbiol Infect 2012 reference 8), although we did not see any seasonality in *S. aureus* infections in our cohort.

Lines 99-102 (Introduction) This part should be mentioned in the discussion or conclusion.

**Response:** This is a very fair comment. We have expanded the discussion accordingly to address the reviewer’s concern about the implications for clinicians managing patients with an undifferentiated presentation (lines 527-536). Public health strategies to address melioidosis, leptospirosis and influenza and already discussed in the body of the discussion (lines 411-419, 427-435 and 514-517).

Lines 108-109 (Materials and methods) Why this disease was selected to be studied, should be dealt with in the introduction section.

**Response:** This is a fair comment. We have expanded the discussion in the introduction and the methods to address the reviewer’s concern (lines 139-164 and Lines 175-178)

Lines 110-111, & 113 (Materials and methods) A detailed description of this database 'Queensland electronic pathology database' should be added.

**Response:** We agree with the reviewer. We have amended the text accordingly (lines 185-186)

Lines 129-138 (Statistical Analysis) As a whole, non-parametric analysis methods were applied. Why?

**Response:** Most of the variables had a non-parametric distribution and so, non-parametric tests (like Kruskal-Wallis were used) were used. We have amended the statistics section to highlight this point (lines 206-207).

Lines 135-136 (Statistical Analysis) Spearman's rho is a calculation method for estimating the correlation coefficient. What do you mean by used to compare continuous variables?

**Response:** The reviewer is correct. We used Spearman's rho to determine the correlation coefficient between the continuous variables (e.g., rainfall and humidity, temperature and rainfall) in Supplementary table 1). We have amended the text of the statistics section to make this clearer (lines 207-208).

Lines 151-152 (Results) " but neither ~ respectively) Please state in a readable way that it is not statistically significant.

**Response:** This is a fair comment. We have amended the text to address the reviewer’s concern.

Lines 153, 215, 221 Please be consistent with putting a thousandth comma in a number.

**Response:** I see from recently published articles in PLOS Global Health that commas were only used for numbers of ≥ 6 digits. We have amended the manuscript accordingly but would be happy to present the data in any way that accords with the Journal’s policy.

Lines 162-163 (Fig 2) There should be an indication for the wet season in Fig 2.

**Response:** This is an excellent suggestion. We have modified the figure (now figure 3) accordingly.

Page 9, Table 2 It seems that the 95% confidence interval in parentheses is, but it is more readable to express it by changing the line rather than spreading it over two lines.

**Response:** This is a very fair comment. In the original submission we did not want the table to go too close to the margin of the page. We have amended the table accordingly.

Pages 229-265 (Discussion) It is not necessary to list by pathogen in the discussion. The text should be written more condense with the main points.

**Response:** We respectfully disagree with the reviewer on this point. The 6 highlighted infections are distinct pathogens and differ - starkly - in their seasonality. Melioidosis and leptospirosis for instance were more common in the wet season, QTT was more common in the dry season, while influenza showed inter-seasonality. The majority of Cryptococcal infections presented in the dry season while the other pathogens (*Staphylococcus aureus* and scrub typhus) showed no seasonality.

As patients are infected by the pathogens in different ways (percutaneously during in the wet season in leptospirosis, percutaneously and by inhalation in the wet season in melioidosis, via vector in the dry season for QTT, by, potentially, inhalation of infective spores during flowering of trees in dry season in the case of Cryptococcus and by inhalation in different seasons in influenza) we felt it best to present the data by individual disease.

In the revised discussion (lines 527-536) we now summarise the main points for the clinician more succinctly, however.

Reviewer #2: "There were 406 cases of leptospirosis during the study period, but no change in annual 194 incidence (p=0.37) (Fig 1). Infections were seasonal with 286 (70.4%) diagnosed in the wet
195 season (p=0.0001) (Fig 2) "
- It is better to report P values smaller than 0.001 as P<0.001

**Response:** This is a very fair comment. We have amended the p values accordingly throughout the text.
